# Heat stress mitigation in tomato (*Solanum lycopersicum* L.) through foliar application of gibberellic acid

**DOI:** 10.1038/s41598-022-15590-z

**Published:** 2022-07-05

**Authors:** Tianxin Guo, Shaista Gull, Muhammad Moaaz Ali, Ahmed Fathy Yousef, Sezai Ercisli, Hazem M. Kalaji, Arkadiusz Telesiński, Alicja Auriga, Jacek Wróbel, Nagy S. Radwan, Rehab Y. Ghareeb

**Affiliations:** 1 College of Horticulture, Fujian Agriculture and Forestry University, Fuzhou, 350002 China; 2 Department of Horticulture, Bahauddin Zakariya University, Multan, 66000 Punjab Pakistan; 3 Department of Horticulture, College of Agriculture, University of Al-Azhar (Branch Assiut), Assiut, 71524 Egypt; 4 Department of Horticulture, Agricultural Faculty, Ataturk University, 25240 Erzurum, Turkey; 5 Department of Plant Physiology, Institute of Biology, Warsaw University of Life Sciences SGGW, Now-oursynowska 159, 02-776 Warsaw, Poland; 6 Institute of Technology and Life Sciences - National Research Institute, Falenty, Al. Hrabska 3, 05-090 Raszyn, Poland; 7 Department of Bioengineering, West Pomeranian University of Technology in Szczecin, 17 Słowackiego Street, 71-434 Szczecin, Poland; 8 Department of Animal Anatomy and Zoology, Faculty of Biotechnology and Animal Husbandry, West Pomeranian University in Szczecin, Janickiego Str. 33, 71-270 Szczecin, Poland; 9 Department of Agricultural Botany, Faculty of Agriculture (Saba Basha), Alexandria University, Alexandria, 21531 Egypt; 10 Plant Protection and Biomolecular Diagnosis Department, Arid Lands Cultivation Research Institute, City of Scientific Research and Technological Applications, Borg El-Arab, 21934 Alexandria Egypt

**Keywords:** Photosynthesis, Plant hormones, Plant physiology, Plant stress responses

## Abstract

Phytohormones mediate physiological, morphological, and enzymatic responses and are important regulators of plant growth and development at different stages. Even though temperature is one of the most important abiotic stressors for plant development and production, a spike in the temperature may have disastrous repercussions for crop performance. Physiology and growth of two tomato genotypes ('Ahmar' and 'Roma') were studied in two growth chambers (25 and 45 °C) when gibberellic acid (GA_3_) was applied exogenously. After the 45 days of planting, tomato plants were sprayed with GA_3_ at concentrations of 25, 50, 75, and 100 mg L^−1^, whereas untreated plants were kept as control. Under both temperature conditions, shoot and root biomass was greatest in 'Roma' plants receiving 75 mg L^−1^ GA_3_, followed by 50 mg L^−1^ GA_3_. Maximum CO_2_ index, photosynthetic rate, transpiration rate, and greenness index were recorded in 'Roma' plants cultivated at 25 °C, demonstrating good effects of GA_3_ on tomato physiology. Likewise, GA_3_ enhanced the proline, nitrogen, phosphorus, and potassium levels in the leaves of both genotypes at both temperatures. Foliar-sprayed GA_3_ up to 100 mg L^−1^ alleviated the oxidative stress, as inferred from the lower concentrations of MDA and H_2_O_2,_ and boosted the activities of superoxide dismutase, peroxidase, catalase. The difference between control and GA_3_-treated heat-stressed plants suggests that GA_3_ may have a function in mitigating heat stress. Overall, our findings indicate that 75 mg L^−1^ of GA_3_ is the optimal dosage to reduce heat stress in tomatoes and improve their morphological, physiological, and biochemical characteristics.

## Introduction

The tomato (*Solanum lycopersicum* L.) is a member of the Solanaceae family, which is native to Peru and Mexico^1,2^. Tomatoes are produced in Pakistan over an area of 58,359 hectares, with an average yearly yield of 550,979 tonnes^3,4^. Tomatoes may be grown in a wide range of climates, although they face a variety of abiotic stresses, including high temperatures^5–7^.

Temperature change has a significant impact on tomato yield^8^. Some physiological processes are inhibited by an increase in optimal temperature, resulting in decreased plant production^9,10^. Heat stress impacts various aspects of plant development, including germination, expansion, and reproduction^11^. High temperatures may cause the photosynthesis apparatus in chloroplasts to malfunction. The major sites of damage owing to high temperature have been identified as carbon metabolism in the stroma and chemical signalling in thylakoid lamellae^12^. Photosynthesis is more heat sensitive as compared to dark respiration and is inhibited before the inhibition of respiration due to the plant's injury caused by high temperature^13,14^. High temperature makes plant tissues lose water, which makes it hard for minerals to get where they need to go^15–19^. When high temperatures stress tomato cultivars, they react in different ways. Up to 10–15% of the crop's yield can be lost for every degree above the optimum temperature^20^.

Technologies and approaches are required to be devised to increase the performance of crops under heat stress. Gibberellic acid (GA_3_), is a plant hormone involved in numerous processes such as plant height, leaf expansion, dry matter accumulation, tissue differentiation, cell division, net absorption rate, blooming, photosynthesis and transpiration rate^21–23^. Furthermore, GA_3_ is a diterpenoid molecule that has been shown to play a vital role in stress resistance in a variety of crops by influencing physiology, morphology, and enzymatic activities^24,25^. Exogenous applications of GA_3_ have been shown in the literature to have a significant impact in *Solanum nigrum* growth and development^25^. Furthermore, foliar GA_3_ treatment resulted in a significant increase in *Carapichea ipecauanha* growth and biomass accumulation compared to untreated plants^26^. Previous studies have linked GA_3_'s protective effect to increased photosynthetic performance^22^. The increased antioxidant activities that decreased oxidative damage in *Corchorus capsularis* L. plants growing under abiotic stress conditions might be the cause for this process^27^. Amino acids and metabolites interact with a variety of biological components, including plant growth regulators, enzymes, polyamines, and nutrients, to create derivatives that are necessary to reduce heat stress^28^. GA_3_ is required for the activation of reactive oxygen species (ROS) scavenging enzymes, which improves antioxidant defense in the case of abiotic stress^29^.

Cultivation is challenging in Pakistan under controlled circumstances due to tiny landholdings, limited resources, and high energy costs^30^. Furthermore, in conventional tomato cultivation systems, high temperatures stress the crop, resulting in low yield and poor fruit quality^8^. As a result, research into the influence of plant growth regulators on tomato heat stress is required. As a result, the current research looked at the effects of exogenously applied GA_3_ as a stress reliever in two distinct tomato cultivars. The GA_3_ was applied at the concentrations of 25, 50, 75, and 100 mg L^−1^ to ‘Roma’ (thermotolerant) and ‘Ahmar’ (thermosensitive) tomatoes grown in two growth chambers (25 and 45 °C).

## Results

### Morphological variables

When compared to all of the other treatments, the untreated plants that were subjected to heat stress at 45 °C had the shortest shoot length (8.37 cm for 'Ahmar' and 14.85 cm for 'Roma'). Not only did the exogenous application of GA_3_ help to alleviate the heat stress, but it also helped to increase the shoot length of both genotypes. When sprayed with 75 mg L^−1^ GA_3_, the plants produced their maximum shoot length under both temperature conditions and in both cultivars. Similarly, plants of 'Roma' treated with 75 mg L^−1^ GA_3_ under heat stress (45 °C) were observed to have the longest roots (13.49 cm), followed by plants receiving normal temperature (25 °C). This finding suggests that 75 mg L^−1^ GA_3_ not only induced thermotolerance but also increased the below ground biomass production of tomato plants (Table 1).

**Table 1:** The length of the tomato shoots and roots, as impacted by temperature, genotype, and exogenous application of GA_3_.

Temperature (A)	Treatment (B)	Shoot length (cm)			Root length (cm)		
		Ahmar	Roma	Mean (A x B)	Ahmar	Roma	Mean (A x B)
25 °C	Control	20.5 ghi	28.01 c-f	24.25 de	5.7 fgh	7.69 d-g	6.69 d
	25 mg L^−1^ GA_3_	20.12 ghi	30.51 b-e	25.31 cde	7.94 def	9.68 bcd	8.81 c
	50 mg L^−1^ GA_3_	19.87 ghi	32.26 a-d	26.06 bcd	7.76 d-g	9.75 bcd	8.75 c
	75 mg L^−1^ GA_3_	22.12 fgh	35.26 ab	28.69 abc	9.77 bcd	11.76 abc	10.77 b
	100 mg L^−1^ GA_3_	15.87 hi	27.01 def	21.44 e	4.85 gh	6.84 d-g	5.84 d
45 °C	Control	8.37 j	14.85 i	11.61 f.	2.96 h	4.93 gh	3.94 e
	25 mg L^−1^ GA_3_	19.76 ghi	32.51 a-d	26.13 bcd	6.03 efg	8.93 cde	7.48 cd
	50 mg L^−1^ GA_3_	24.51 efg	34.26 abc	29.38 ab	9.65 bcd	11.75 abc	10.7 b
	75 mg L^−1^ GA_3_	22.51 fg	38.26 a	30.38 a	12.34 ab	13.49 a	12.91 a
	100 mg L^−1^ GA_3_	18.26 ghi	29.01 b-e	23.63 de	5.03 fgh	7.08 d-g	6.06 d
Mean (genotype)		19.19 b	30.19 a		7.2 b	9.19 a	
HSD_0.05_ (Interaction)			6.378			2.951	

According to Tukey's honestly significant difference test, the same letters suggest that there is no statistically significant difference between treatments (*p* ≤ 0.05).

‘Roma’ being a thermotolerant cultivar experienced better results than ‘Ahmar’ in terms of biomass accumulation. In case of shoot fresh weight of tomato, the plants treated with 75 mg L^−1^ GA_3_ showed maximum values in both temperature conditions followed by 50 mg L^−1^, 25 mg L^−1^, and 100 mg L^−1^ GA_3_ application. Similar to the aforementioned variables, ‘Roma’ (36.93 g) showed better shoot fresh weight than ‘Ahmar’ (31.51 g). Tomato plants that were subjected to heat stress at a temperature of 45 °C had the lowest possible shoot fresh weight until GA_3_ was sprayed. Both at 45 °C (42.14 g 'Ahmar' and 46.65 g 'Roma') and at 25 °C (43.32 g 'Ahmar' and 46.11 g 'Roma'), the plants that were treated with 75 mg·L^−1^ GA_3_ had the fresh shoots with the maximum weight. A similar pattern was seen in terms of shoot dry weight. The plants receiving 75 mg L^−1^ GA_3_ showed maximum values in both temperature conditions followed by 50 mg L^−1^, 25 mg L^−1^, and 100 mg L^−1^ GA_3_ application. ‘Roma’ (11.19 g) showed better shoot dry weight than ‘Ahmar’ (9.39 g). The plants treated with 75 mg L^−1^ GA_3_ exhibited maximum shoot dry weight at 45 °C (13.16 g ‘Ahmar’; 14.57 g ‘Roma’) as well as 25 °C (13.53 g ‘Ahmar’; 14.40 g ‘Roma’) (Table 2).

**Table 2:** Shoot fresh and dry weight of tomato as affected by temperature, genotype and exogenous application of GA_3_.

Temperature (A)	Treatment (B)	Shoot fresh weight (g)			Shoot dry weight (g)		
		Ahmar	Roma	Mean (A x B)	Ahmar	Roma	Mean (A x B)
25 °C	Control	21.66 hij	28.2 fgh	24.93 e	6.76 gh	8.81 ef	7.79 de
	25 mg L^−1^ GA_3_	30.39 fgh	34.68 c-g	32.53 cd	9.49 def	10.83 cde	10.16 c
	50 mg L^−1^ GA_3_	34.55 c-g	41.34 a-e	37.94 bc	10.79 cde	12.91 ab	11.85 b
	75 mg L^−1^ GA_3_	43.32 abc	46.11 ab	44.71 a	13.53 a	14.4 a	13.97 a
	100 mg L^−1^ GA_3_	26.63 ghi	33.42 d-g	30.02 de	5.82 hi	8.94 ef	7.38 e
45 °C	Control	14.24 j	18.74 ij	16.49 f.	4.45 i	5.85 hi	5.15 f.
	25 mg L^−1^ GA_3_	34.13 c-g	40.72 a-e	37.42 bc	10.66 de	12.72 abc	11.69 b
	50 mg L^−1^ GA_3_	35.91 c-f	42.52 a-d	39.21 ab	11.22 bcd	13.28 a	12.25 b
	75 mg L^−1^ GA_3_	42.14 a-d	46.65 a	44.39 a	13.16 ab	14.57 a	13.87 a
	100 mg L^−1^ GA_3_	32.15 efg	36.96 b-f	34.55 bcd	8.04 fg	9.55 def	8.79 d
Mean (genotype)		31.51 b	36.93 a		9.39 b	11.19 a	
HSD_0.05_ (Interaction)			9.217			2.043	

According to Tukey's honestly significant difference test, the same letters suggest that there is no statistically significant difference between treatments (*p* ≤ 0.05).

In contrast to the previously reported variable, tomato plants of the 'Ahmar' cultivar that were given 75 mg L^−1^ of GA_3_ had the highest value of root fresh weight (12.21 g). Both of these temperature circumstances brought out the best in the 'Roma' cultivar plants, which were treated with a foliar treatment of 50 mg L^−1^ of GA_3_. The largest value of root dry weight (6.44 g) was reported in plants of the ‘Roma' cultivar that had been treated with 75 mg L^−1^ GA_3_ at room temperature (25 °C) throughout the experiment (Table 3).

**Table 3:** Root fresh and dry weight of tomato as affected by temperature, genotype and exogenous application of GA_3_.

Temperature (A)	Treatment (B)	Root fresh weight (g)			Root dry weight (g)		
		Ahmar	Roma	Mean (A x B)	Ahmar	Roma	Mean (A x B)
25 °C	Control	5.82 def	7.81 b-e	6.82 cd	2.26 efg	2.98 cde	2.62 cd
	25 mg L^−1^ GA_3_	7.57 b-e	9.56 a-d	8.57 bc	2.9 cde	4.36 bcd	3.63 bc
	50 mg L^−1^ GA_3_	9.65 abc	11.64 a	10.64 ab	2.44 ef	4.43 bcd	3.43 c
	75 mg L^−1^ GA_3_	7.64 b-e	9.63 abc	8.63 bc	4.45 bcd	6.44 a	5.44 a
	100 mg L^−1^ GA_3_	4.72 ef	6.71 b-f	5.72 d	0.6 g	1.51 efg	1.06 e
45 °C	Control	3.55 f.	6.05 c-f	4.8 d	1.11 fg	1.53 efg	1.32 e
	25 mg L^−1^ GA_3_	7.66 b-e	9.8 abc	8.73 abc	2.77 def	4.6 bc	3.69 bc
	50 mg L^−1^ GA_3_	9.53 a-d	11.63 a	10.58 ab	4.33 bcd	4.93 ab	4.63 ab
	75 mg L^−1^ GA_3_	12.21 a	9.87 ab	11.04 a	5.26 ab	5.92 ab	5.59 a
	100 mg L^−1^ GA_3_	4.91 ef	6.95 b-f	5.93 d	1.56 efg	1.75 efg	1.66 de
Mean (genotype)		7.33 b	8.97 a		2.77 b	3.84 a	
HSD_0.05_ (Interaction)			3.76			1.783	

According to Tukey's honestly significant difference test, the same letters suggest that there is no statistically significant difference between treatments (*p* ≤ 0.05).

### Physiological variables

**Figure 1:**
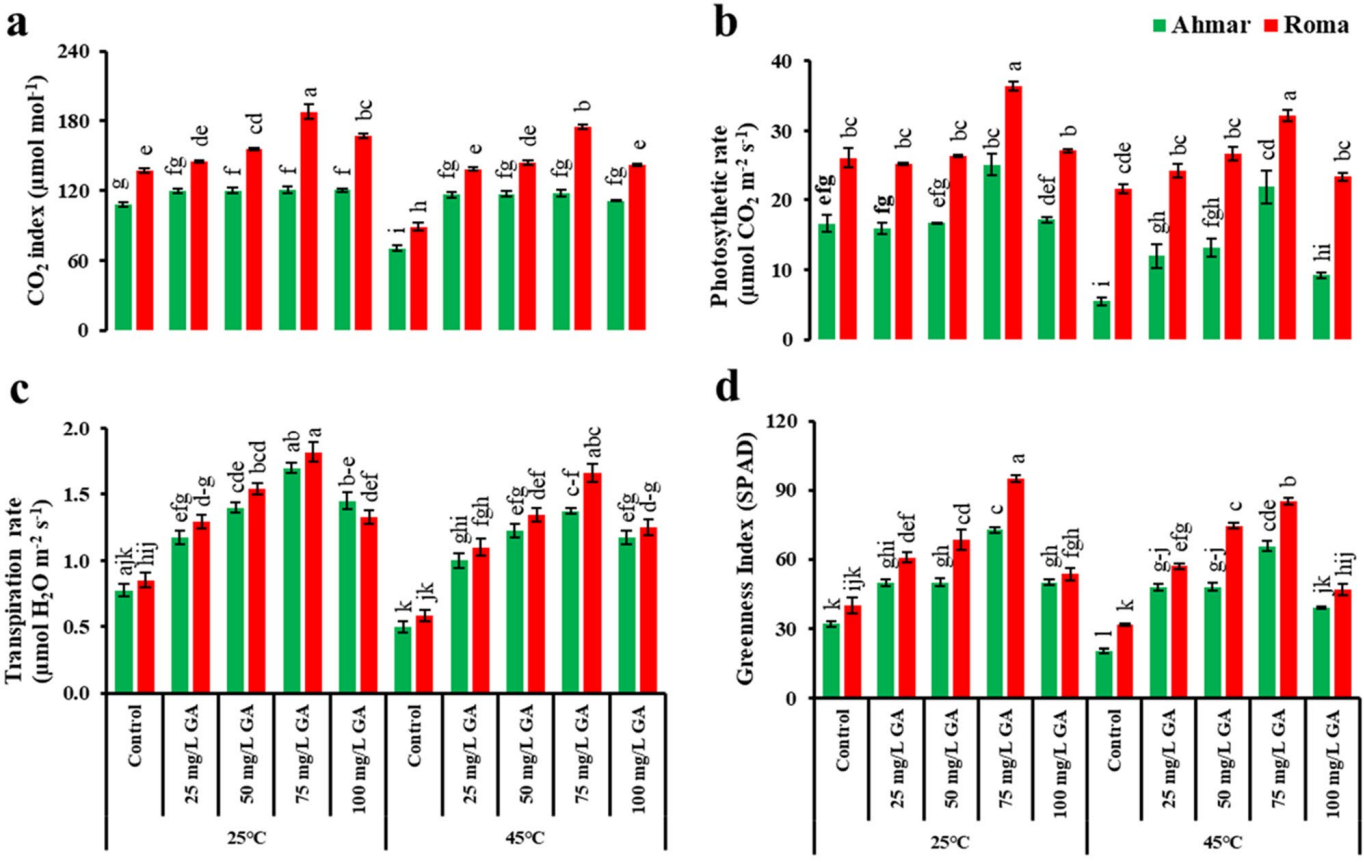
Physiological variables of tomato as affected by temperature, genotype and exogenous application of GA_3_. According to Tukey's honestly significant difference test, the same letters suggest that there is no statistically significant difference between treatments (*p* ≤ 0.05). Vertical bars indicate average ± standard error (n = 4, 5 plants per replicate).

In general, the findings that are shown in Fig. 1 suggest that 'Roma,' which is a heat-resistant cultivar, had superior physiologic properties in comparison to 'Ahmar not only when the plants were subjected to heat stress but also when the temperatures were at normal levels. To be more specific, the tomato plants (cv. 'Roma') treated with 75 mg L^−1^ GA_3_ showed maximum CO_2_ index, photosynthetic rate, transpiration rate, and greenness index under normal temperature (25 °C) followed by heat stress (45 °C). Their values were as follows: 188.1 µmol mol^−1^, 36.3 µmol CO_2_ m^−2^ s^−1^, 1.8 µmol H_2_O m^−2^ s^−1^, and 95 SPAD, respectively. When the exogenous application of GA_3_ was performed on tomato plants (cv. 'Ahmar'), the CO_2_ index rose regardless of the concentration that was used. This was seen at both temperature conditions (Fig. 1a).

Because it is a thermosensitive cultivar, 'Ahmar' demonstrated a drop in photosynthetic rate of control plants under heat stress that was 2.2 times greater than the loss in photosynthetic rate seen in plants maintained at the optimum temperature. However, the application of 75 mg L^−1^ GA_3_ resulted in a considerable increase in photosynthetic rate when compared to the control (5.5 µmol CO_2_ m^−2^ s^−1^ ‘Ahmar’; 21.6 µmol CO_2_ m^−2^ s^−1^ ‘Roma’). This was the case for both 'Ahmar' and 'Roma' (Fig. 1b). With the foliar application of GA_3_, the transpiration rate of tomato plants (both cultivars 'Ahmar' and 'Roma') significantly increased. In both growth chambers, maximum transpiration rate was exhibited by the plants (Cv. ‘Roma’) treated with 75 mg·L^−1^ GA_3_ (1.8 µmol H_2_O m^−2^ s^−1^ ‘25 °C’, 1.7 µmol H_2_O m^−2^ s^−1^ ‘45 °C) (Fig. 1c). In a similar manner, plants of the 'Roma' cultivar that had foliar application of 75 mg·L^−1^ GA_3_ shown an increase in greenness index of 137 and 168%, when subjected to temperatures of 25 °C and 45 °C, respectively. However, the 'Ahmar' cultivar plants that were given 75 mg L^−1^ GA_3_ showed an increase in greenness index that was 127% higher at 25 °C and 224% at 45 °C. Despite the fact that 'Ahmar' was a heat-sensitive cultivar, it showed significantly improved results when it was given an exogenous treatment of GA_3_ (Fig. 1d).

### Biochemical variables

**Figure 2:**
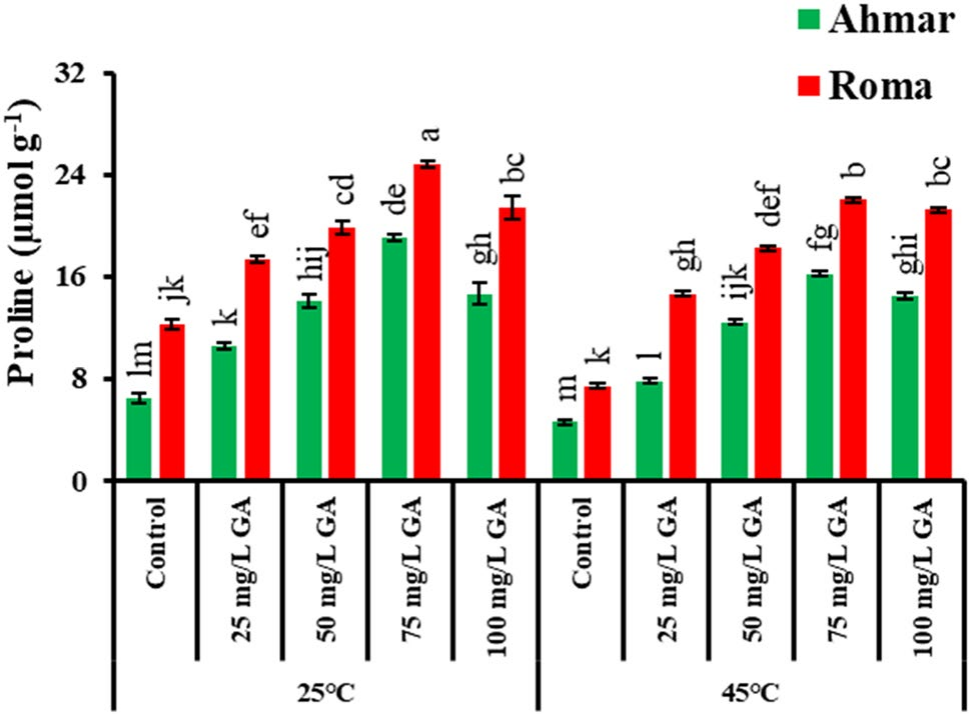
Proline content of tomato as affected by temperature, genotype and exogenous application of GA_3_. According to Tukey's honestly significant difference test, the same letters suggest that there is no statistically significant difference between treatments (*p* ≤ 0.05). Vertical bars indicate average ± standard error (n = 4, 5 plants per replicate).

Tomato plants (cv. ‘Roma’) treated with 75 mg L^−1^ GA_3_ showed maximum leaf proline content (24.8 µmol g^−1^) under normal temperature (25 °C) followed by heat stress (45 °C). The amount of proline in the leaves rose in a dose-dependent manner in response to the application of GA_3_ when the plants were subjected to heat stress (Fig. 2).

**Figure 3:**
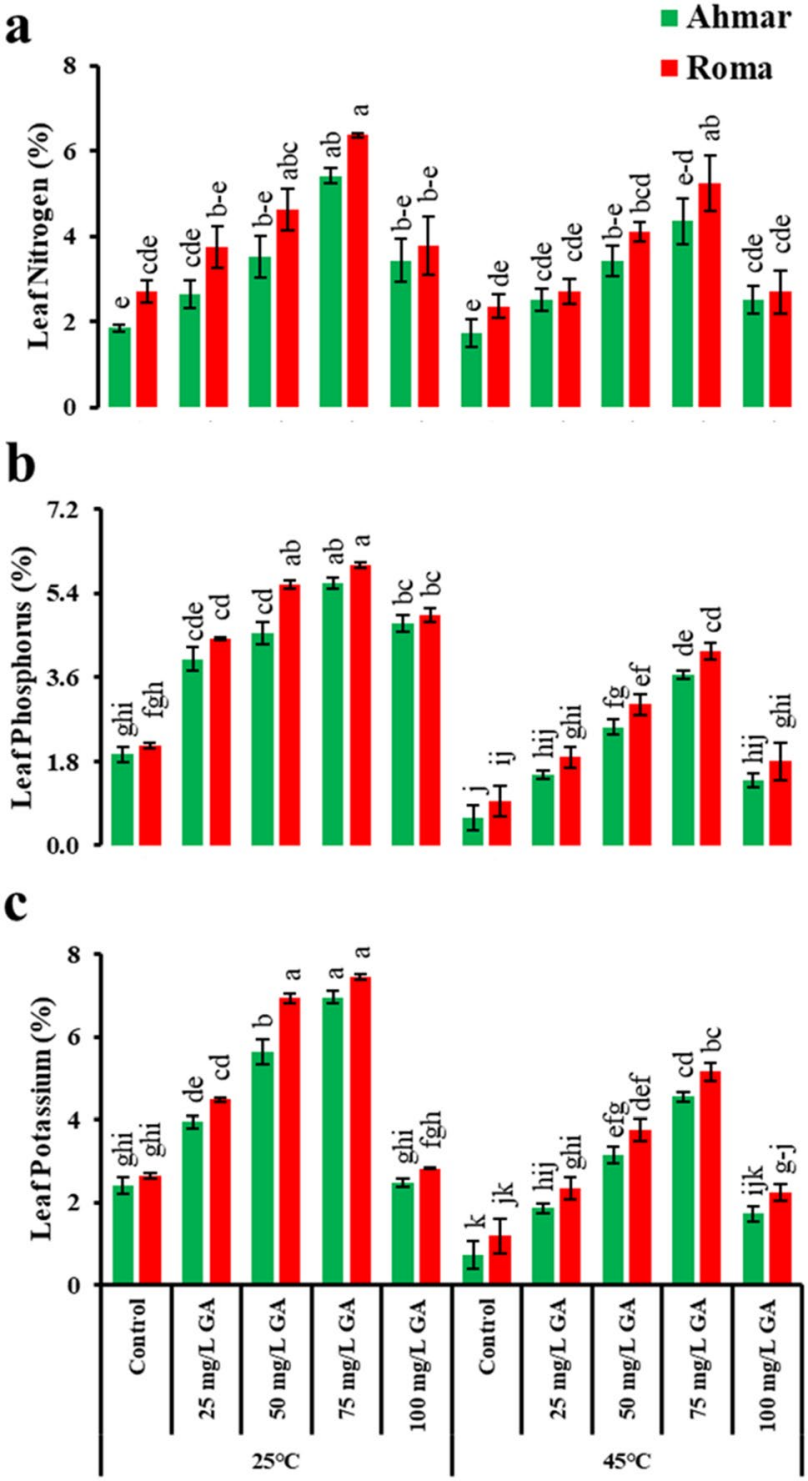
Leaf minerals concentration of tomato as affected by temperature, genotype and exogenous application of GA_3_. According to Tukey's honestly significant difference test, the same letters suggest that there is no statistically significant difference between treatments (*p* ≤ 0.05). Vertical bars indicate average ± standard error (n = 4, 5 plants per replicate).

Similarly, tomato plants (cv. ‘Roma’) treated with 75 mg L^−1^ GA_3_ showed maximum leaf contents of nitrogen, phosphorus, and potassium (6.4%, 6%, and 7.4%, respectively) under normal temperature (25 °C) followed by heat stress (45 °C). In case of leaf N level, plants of both cultivars showed non-significant difference among each other except the plants treated with 75 mg L^−1^ GA_3_ under heat stress (Fig. 3a). Similarly, leaf P and K level remained unchanged between cultivars (except when 50 mg L^−1^ GA_3_ was applied) but significantly increased with the exogenous application of GA_3_. ‘Ahmar’ being a thermosensitive cultivar showed a 3.3 and 3.5-fold decrease in phosphorus and potassium level, respectively, under heat stress as compared to the plants grown under normal temperature (Fig. 3b,c).

### Oxidative stress indicators and antioxidant response

**Figure 4:**
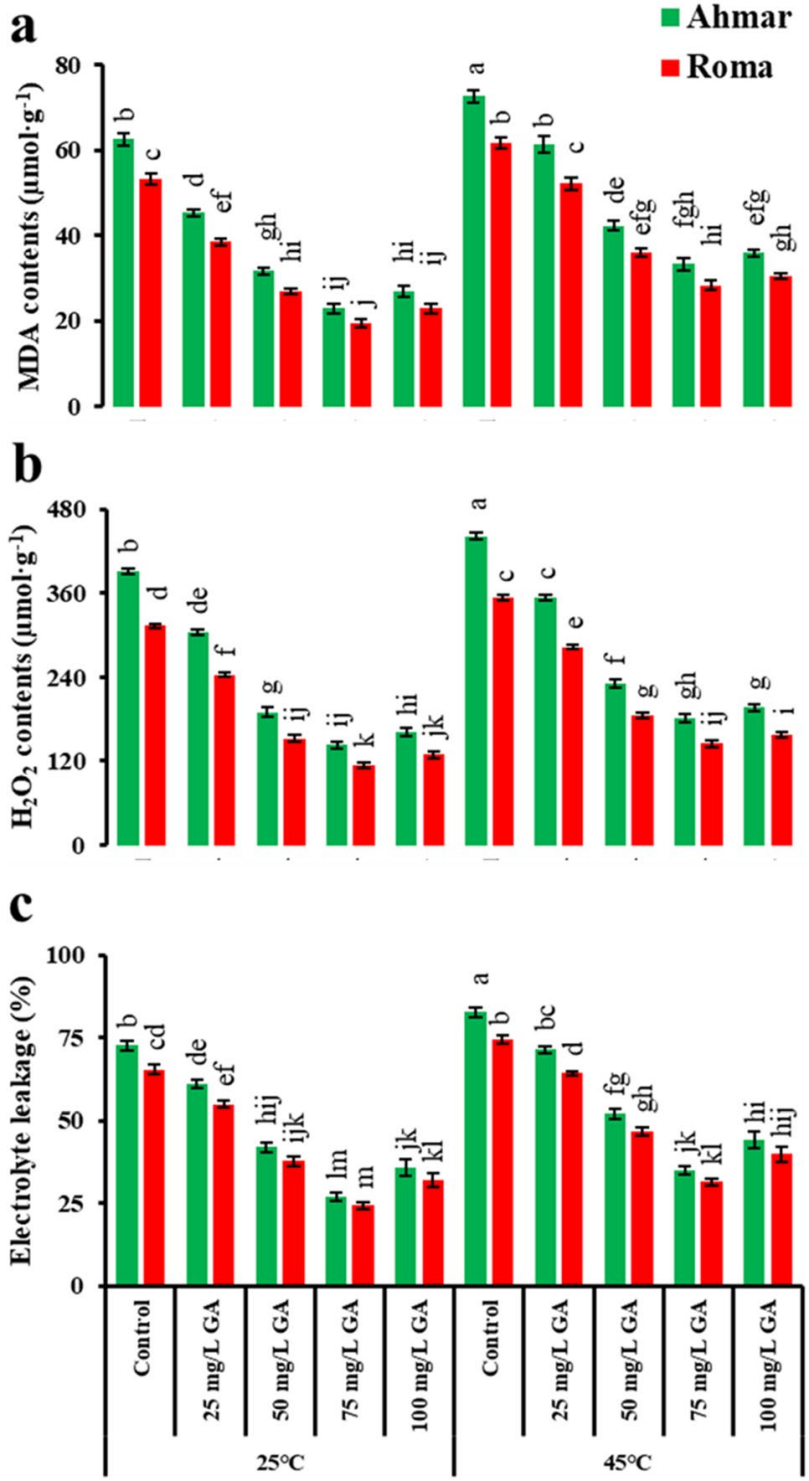
Oxidative stress indicators of tomato as affected by temperature, genotype and exogenous application of GA_3_. According to Tukey's honestly significant difference test, the same letters suggest that there is no statistically significant difference between treatments (*p* ≤ 0.05). Vertical bars indicate average ± standard error (n = 4, 5 plants per replicate).

Plants grown under normal temperature (25 °C), when treated with 75 mg L^−1^ GA_3_ showed minimum MDA and H_2_O_2_ contents and electrolyte leakage (23 µmol·g^−1^, 143.32 µmol g^−1^ and 27.2%, respectively for ‘Ahmar’, and 19.55 µmol g^−1^, 114.66 µmol g^−1^ and 24.3%, respectively for ‘Roma’). The plants grown under heat stress (45 °C) exhibited increased electrolyte leakage, MDA and H_2_O_2_ contents than those were grown under normal temperature. The exogenous application of GA_3_ significantly reduced electrolyte leakage, MDA and H_2_O_2_ contents in concentration-dependent manner. The maximum decrease in MDA, H_2_O_2_ and electrolyte leakage were observed in plants treated with 75 mg L^−1^ GA_3_ as compared to other experimental units and control (Fig. 4).

**Figure 5:**
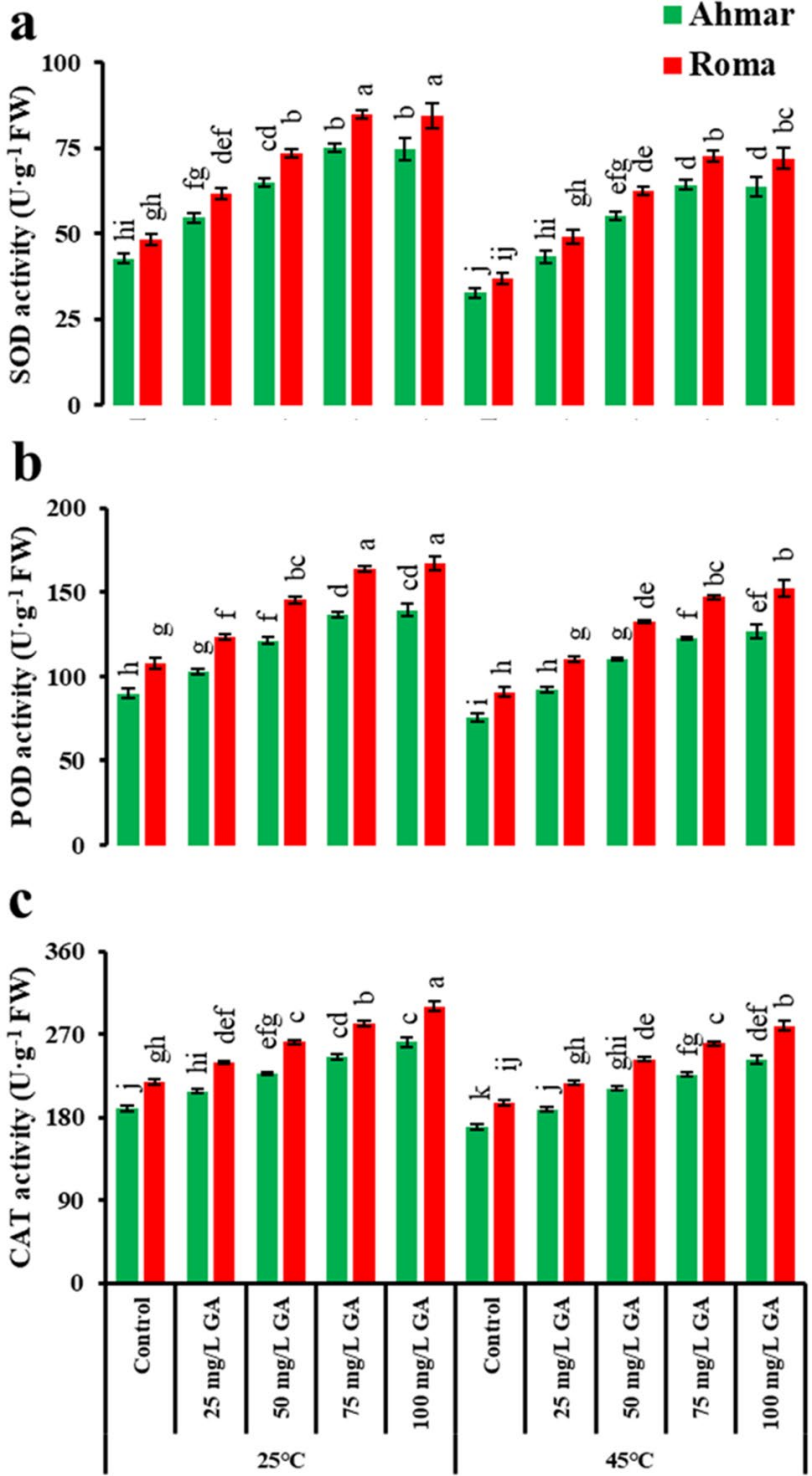
Activities of antioxidant enzymes in tomato as affected by temperature, genotype and exogenous application of GA_3_. According to Tukey's honestly significant difference test, the same letters suggest that there is no statistically significant difference between treatments (*p* ≤ 0.05). Vertical bars indicate average ± standard error (n = 4, 5 plants per replicate).

The exogenous application of 75 mg L^−1^ GA_3_ exhibited maximum SOD activity in the plants grown under normal temperature (75 U g^−1^ FW ‘Ahmar’; 84.75 U g^−1^ FW ‘Roma’) followed by the plants grown under heat stress (64.39 U g^−1^ FW ‘Ahmar’; 72.69 U g^−1^ FW ‘Roma’). In similarity with the aforementioned variable, the highest POD activity (139.5 U·g^−1^ FW ‘Ahmar’; 167.4 U g^−1^ FW ‘Roma’) was also observed in tomato plants grown under normal temperature (25 °C) treated with 100 mg L^−1^ GA_3_. Plants receiving the foliar application of 75 mg L^−1^ GA_3_ also showed better performance in both temperature conditions. In the case of CAT activity, maximum values (261.35 U g^−1^ FW ‘Ahmar’; 300.55 U g^−1^ FW ‘Roma’) were also recorded in plants treated with 100 mg L^−1^ GA_3_ under normal temperature conditions. The reduced activity of antioxidant enzymes i.e., SOD, POD and CAT in untreated plants grown under heat stress indicates a significant effect of heat stress on tomato plants (Fig. 5).

### Correlation analysis

**Figure 6:**
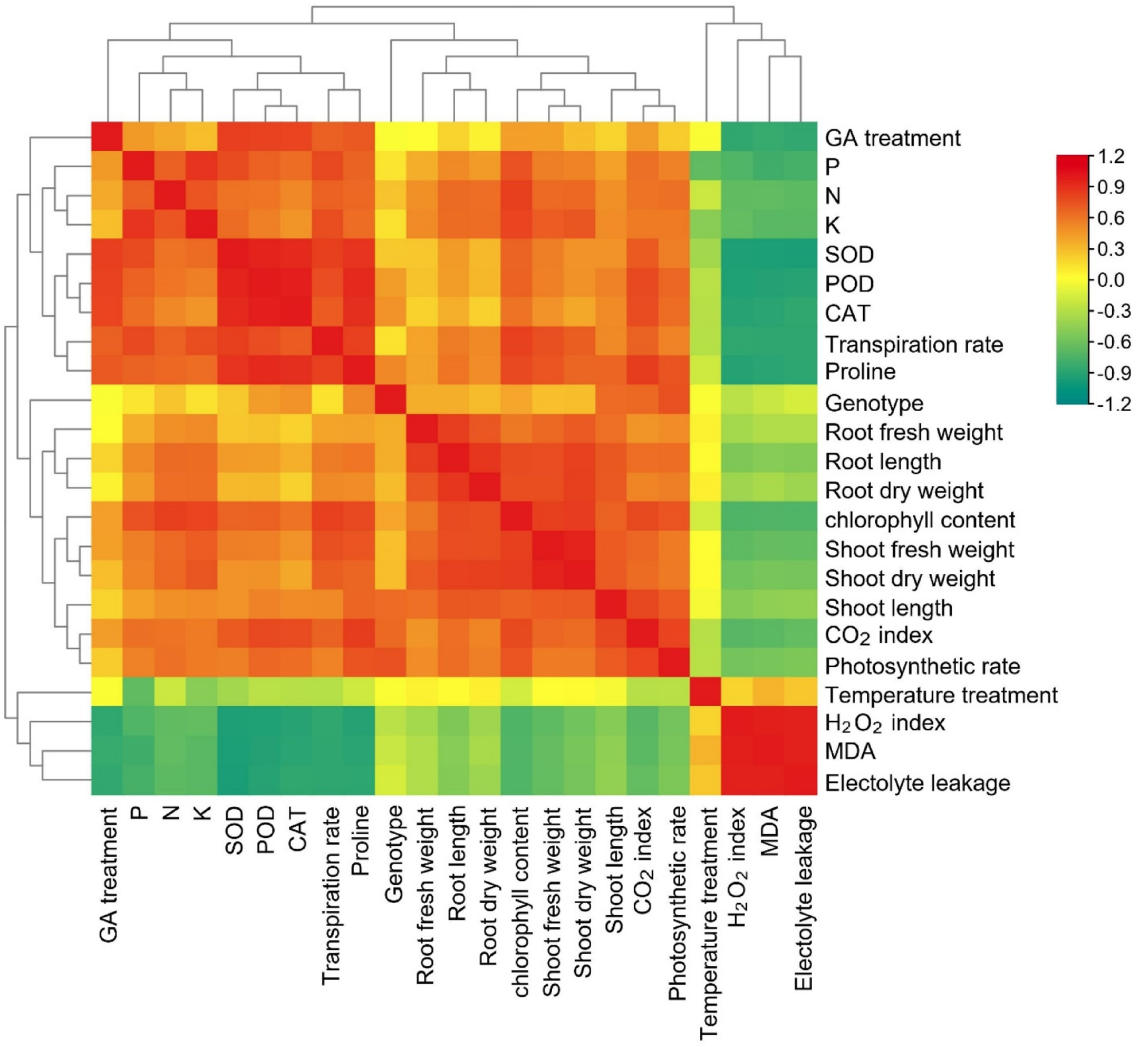
Correlation analysis among GA_3_ treatments and various morphological, physiological, and biochemical variables of tomato cv. ‘Ahmar’ and ‘Roma’ under heat stress.

Pearson (*n*) correlation analysis was conducted to between GA_3_ treatments and various morphological, physiological, biochemical and antioxidant variables of tomato cv. ‘Ahmar’ and ‘Roma’ under heat stress (Fig. 6). The correlation analysis indicated that tomato genotype showed strong positive correlation with shoot and root length, shoot fresh and dry weight, root fresh and dry weight, CO_2_ index, photosynthesis rate, leaf chlorophyll content, proline, leaf N, SOD, POD and CAT activity, when *p* ≤ 0.05. Similarly, temperature treatments were positively associated with CO_2_ index, photosynthesis rate, transpiration rate, leaf N, P and K, MDA contents, H_2_O_2_ index, and electrolyte leakage. The gibberellic acid treatments were positively and significantly (*p* ≤ 0.05) correlated with shoot and root length, shoot fresh and dry weight, CO_2_ index, photosynthesis rate, transpiration rate, leaf chlorophyll content, proline, leaf N, P and K, SOD, POD and CAT activity. All the tested morphological, physiological, biochemical and antioxidant variables were significantly (*p* ≤ 0.001) correlated to each other.

## Discussion

High temperatures have a variety of effects on plant growth and development. The movement of the cyclin-dependent kinase enzyme, which is decreased as the temperature rises, regulates plant biomass accumulation^31^. The current research found that heat stress had a significant impact on the length and fresh and dry weight of shoots and roots. The cultivar 'Roma,' on the other hand, was unaffected and maintained biomass (Tables 1, 2, 3). Another explanation for reduced biomass accumulation is an increase in senescence caused by hot temperatures. Due to accelerated senescence at high temperatures, maize and wheat plants produced decreased biomass and yield^32,33^.

Gibberellic acid was used as a foliar treatment to alleviate heat stress in tomato plants in this research. In comparison to other GA_3_ treatments and the control (Tables 1, 2, 3), plants treated with 75 mg·L^−1^ GA_3_ accumulated the most biomass, demonstrating that GA_3_ has a favorable function in boosting plant development and alleviating the effects of heat stress. Our findings are consistent with those of Chen et al.^34^, who found that applying GA_3_ to *Vigna radiata* boosted biomass. In Arabidopsis, exogenous administration of GA_3_ was shown to restore the fatal effects of salt, heat, and oxidative stress^35^. According to Khan et al.^36^, exogenous GA_3_ treatment was more effective in reducing high temperature stress in date palms by considerably increasing plant height and fresh, dry biomass weight.

Various abiotic stresses, such as buildup of biomass, chlorophyll, minerals, gas exchange, electrolyte leakage, and the activity of reactive oxygen species, are lessened by gibberellins, which promote plant development while also alleviating their inhibitory effects^35,37,38^. Light-dependent reactions in photosynthesis are influenced by chlorophyll quantity in plants, according to Lüttge^39^. Increased synthesis of antioxidants in chloroplast has been shown to remove reactive oxygen species (ROS) and reduce oxidative damage to photosynthetic membranes^27^. The GA_3_ had a considerable impact on chlorophyll content and gas exchanges, as shown by our results (Fig. 1).

Tomatoes are sensitive to changes in temperature, which may have a significant negative impact on the plant's physiology and growth^40^. The primary factor contributing to reduced plant development is a slowdown in the pace at which photosynthetic reactions take place, which disrupts the operation of mitochondria^41^. According to the findings of our research, tomato plants exposed to heat had a lower rate of net photosynthesis when compared to plants that had been cultivated at temperatures that were considered to be normal (Fig. 1b). Rubisco synthesis (Calvin cycle) is regarded to be a vital phase in photosynthesis, and it was inhibited at temperatures between 35 and 40 °C, resulting in lower net photosynthetic adaption and carbohydrate production^42^. In comparison to plants that were cultivated at ambient temperature and treated with foliar sprays of GA_3_, those that were subjected to heat stress at 45 °C had a lower CO_2_ index (Fig. 4a). During heat stress, mesophyll cells were extensively injured and the permeability of the plasma membrane was enhanced, which resulted in a reduction in stomatal conductance in grapes^43^.

Under heat stress, browning of leaves and stems, slowed growth, leaf abscission, and short length of roots and shoots are some of the macroscopic manifestations of physiological damage that may be detected^44,45^. Heat stress induces an abrupt increase in the rate of transpiration, which in turn leads to dehydration of the organs and a restriction in development^46,47^. It also impacts the rate of photosynthesis and transpiration, as well as the absorption and translocation of water, ions, and entire solutes across the plant membranes^48^. The breakdown of chlorophyll pigmentation is caused by a reduction in photosynthesis rate, which in turn leads to inhibition of photosystem II (PSII)^49,50^. As a further consequence of heat stress, there was a diminishment in the greenness index of tomato leaves (Fig. 1d). The thylakoid membrane may be disrupted by heat stress, which can lead to a reduction in chlorophyll concentration^51–53^. The provision of adequate nutrition to plants leads to an enhancement of photosynthesis via an increase in the production of chlorophyll and plays a role in the expansion and maturation of plant life^54^. In addition to this, it has a significant impact on the function of the tomato plant's xylem and phloem by reducing the amount of mineral transfer^55^.

In the current experiment, heat stress decreased the nitrogen, phosphate, potassium, and proline levels of the leaves, while plants that received foliar spray of GA_3_ not only maintained but also enhanced their nutrition (Figs. 2, 3). Changes in the mineral nutrient content of the soil are directly connected to alterations in the physiological response of the plant^56^. Gibberellic acid has a connection that is synergistic with nitrogen, phosphorus, and potassium, and it stimulates the maximal absorption of these nutrients in plants, which leads to increased plant growth^29^. In addition to this, it has a profound connection to the absorption of nitrogen.

The effectiveness of GA_3_ in modulating plant physiology is dependent on the concentration of the GA_3_, the manner by which it is applied, and the genetics of the plant^57,58^. The findings of this research also demonstrated that the reaction of tomato plant growth and development to the application of GA_3_ varied depending on the concentration of the GA_3_ used. In general, the findings revealed that GA_3_ stimulated the development of tomato plants despite the presence of heat stress. Application of GA_3_ by foliar spray at a concentration of 75 mg·L^−1^ was shown to have a favourable correlation with the morphological, physiological, and biochemical characteristics of tomato.

## Materials and methods

### Experimental site and conditions

An experiment was conducted under controlled conditions at Samundri, Faisalabad, Pakistan (31°07′57.8"N 73°02′03.5"E) from 15 March 2021 to 30 May 2021. Vegetable Research Institute, Ayyub Agriculture Research Institute, located in Faisalabad 38000, Punjab, Pakistan, provided the researchers with seeds that were three months old and came from two different tomato genotypes: 'Roma' (thermotolerant) and 'Ahmar' (thermosensitive)^4,59,60^. Prior to planting, the moisture content of the seeds for 'Ahmar' and 'Roma' was 11% and 10%, respectively. The seeds were planted in plastic pots (33 × 30 cm) containing 12 kg of porous soil obtained from an adjacent field. The structural type of the soil was sandy loam, and its electric conductivity and pH were measured to be 0.401 dS m^−1^ and 6.9, respectively. The EC meter (HI-98304, Hanna Instruments Inc., Mauritius) and the digital pH meter (Hanna, HI-98107, Mauritius) were used to record the electric conductivity and pH, respectively. There were five seeds planted in each pot, and there were five pots that made up each replication. By monitoring the level of moisture in the rooting medium, appropriate amounts of water were added to the pots so that the plants received what they need. Hoagland's solution [0.4 NH_4_H_2_PO_4_; 2.4 KNO_3_; 1.6 Ca(NO_3_)_2_; 0.8 MgSO_4_; 0.1 Fe as Fe-chelate; 0.023 B as B(OH)_3_ [boric acid]; 0.0045 Mn as MnCl_2_; 0.0003 Cu as CuCl_2_; 0.0015 Zn as ZnCl_2_; 0.0001 Mo as MoO_3_ or (NH_4_)_6_Mo_7_O_24_; Cl as chlorides of Mn, Zn, and Cu (all concentrations in units of μM/L)] was used for plants fertigation. The experiment was planned using a split-split plot design, with temperature serving as the main-plot factor, genotypes serving as the sub-plot factor, and GA_3_ treatments serving as the sub-subplot factor, with four repetitions.

**Figure 7:**
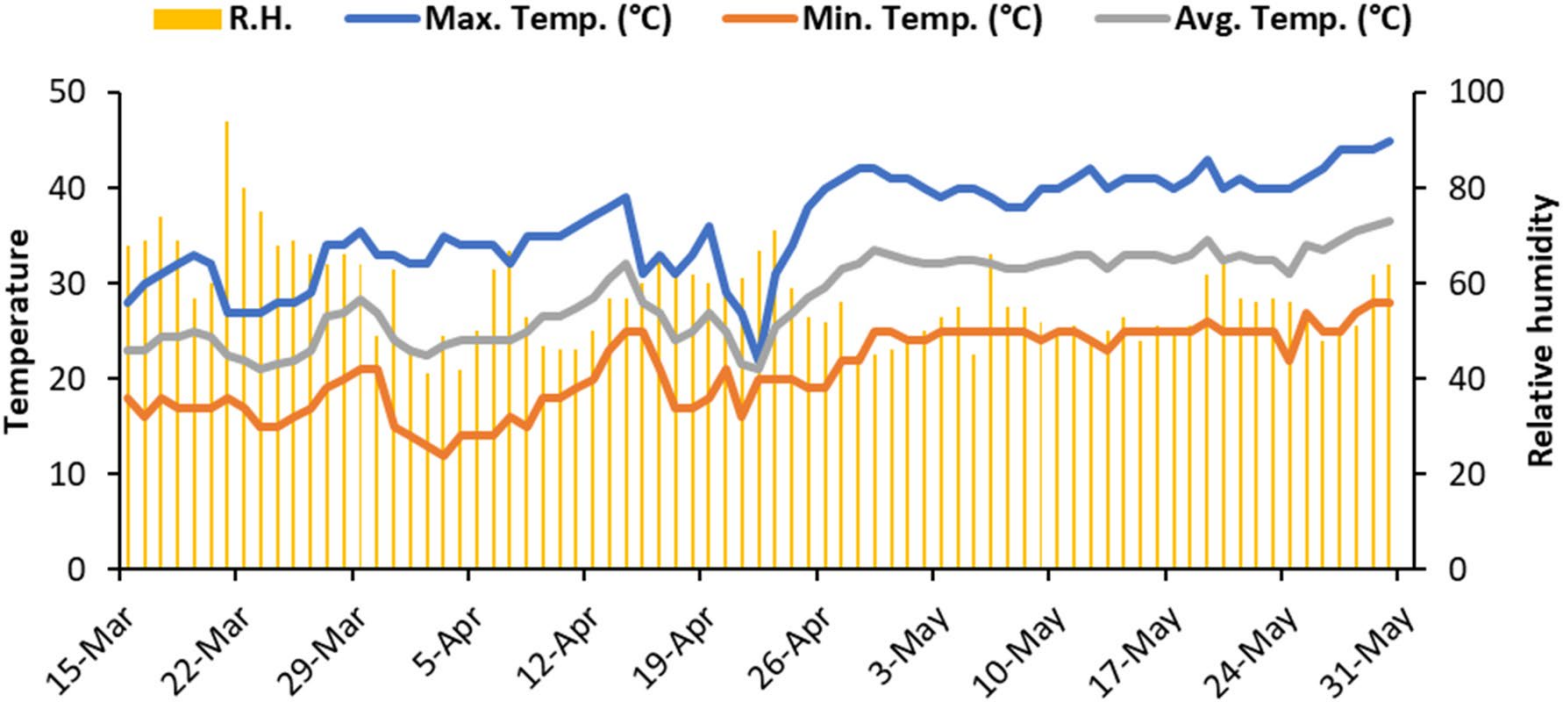
Weather conditions during the experiment^61^.

Although the experiment was conducted under controlled conditions, the environmental data of the region about temperature and relative humidity was obtained (Fig. 7). During the experiment, the average mean temperature was 28.5 °C, with a sharp decrease from 25 to 21 °C (on 23 March and 22 April, respectively), whereas minimum and maximum temperatures oscillated between 12–28 and 22–45 °C, respectively. The average relative humidity varied between 41 and 94%, with the lowest value recorded at 02 April and highest one at 21 March, 2021 (Fig. 7).

### Treatments

Plants of both genotypes were kept in two separate growth chambers (Jeiotech GC-300TL, Scientific Laboratory Supplies, UK). Temperature of both growth chambers was maintained at 25 °C during the day and 20 °C at night with a light period of 12 h [100 ± 2 μmol m^−2^ s^−1^ white florescent light peak wavelength λp (544 nm)]. Following an initial growth period of four weeks, the plants began receiving heat treatments. To prevent osmotic shock, the temperature in one growth chamber was raised by 2 °C every day until the target temperature (45 ± 2 °C during the day and 35 ± 2 °C at night) was reached. The growth chamber experiment was carried out at a relative humidity of 65 ± 5% the whole time. Different levels of GA_3_ (CAS no. 77-06-5, ≥ 90% purity, Sigma-Aldrich Solutions, Darmstadt, Germany) (25, 50, 75, and 100 mg·L^−1^) were applied twice (15 and 22 days after heat induction) through foliar spray in both growth chambers. Control plants were sprayed with water only.

### Morphological variables

Thirty days following the GA_3_ treatment, we examined morphological characteristics of tomato plants. Five randomly chosen plants from each replication were measured using a meter rod, and the average length of shoot and root was determined. A computerized weighing balance was used to weigh fresh shoots and roots (MJ-W176P, Panasonic, Japan). Shoots and roots were oven-dried at 70 °C (YH-9203A, Qingdao Yosion Labtech Co. Ltd., China) until they attained a consistent weight for the purpose of determining dry weights^4,62,63^.

### Physiological variables

Plant physiological variables, i.e., CO_2_ index (µmol mol^−1^), photosynthetic rate (µmol CO_2_ m^−2^ s^−1^), and transpiration rate (µmol H_2_O m^−2^ s^−1^) were measured through LCA-4 infrared gas analyzer (ADC BioScientific Ltd., Hoddesdon, UK) from fully expanded leaves 25 days after GA_3_ application. The leaves greenness index was measured with a chlorophyll SPAD meter (CCM-200 plus, Opti-Sciences, Hudson, NH, USA) according to manufacturer’s instructions, and presented as SPAD values.

### Biochemical variables

Fully expanded, mature, and healthy leaves along with petiole were collected from randomly selected plants from each replicate 25 days after GA_3_ application. Estimation of nitrogen, phosphorus, and potassium in leaf tissues were carried out through micro Kjeldahl’s apparatus, spectrophotometer and flame photometer, respectively, as described by Estefan et al.^64^. Proline concentration was determined through the method of Bates et al.^65^ using spectrophotometer. Fresh leaf tissues (0.5 g) were homogenized in 10 ml of 3% sulfosalicylic acid. The 2 ml filtered homogenate was taken in a test tube and 2 ml acid ninhydrin solution (1.25 g ninhydrin in 30 ml glacial acetic acid and 20 ml 6 M ortho-phosphoric acid) along with 2 ml of glacial acetic acid was added, and heated for 1 h at about 100 °C. Reaction was finished in an ice bath. Reaction mixture was removed with 10 ml toluene, mixed dynamically by passing an incessant stream of air for 1–2 min. Toluene was aspirated from chromophore. Aqueous phase was taken and absorbance was observed at 520 nm using toluene as a blank. Proline concentration was evaluated from a standard curve and analyzed on fresh weight basis as follows:

1 $$ {\text{Proline}}(\mu {\text{molg}}^{{ - 1}} ) = \frac{{{\text{Proline}}\left( {\frac{{\text{g}}}{{{\text{ml}}}}} \right){\text{Xtoluene}}({\text{ml}})}}{{{\text{leafsample}}({\text{g}})}} $$

### Oxidative stress indicators and antioxidant response

To determine malondialdehyde (MDA) content, indicator of lipid peroxidation, 0.1 g leaves were ground with 25 mL of 50 mM phosphate buffer solution containing 1% polyethylene pyrrole with the help of pestle and mortar. After centrifugation at 12,000×*g* for 15 min at 4 °C, the supernatant was taken followed by heating at 100 °C for 20 min. The tubes were quickly cooled in an ice bath after heating. The absorbance was taken at wavelengths of 532, 600 and 450 nm by using a spectrophotometer (T60 U Spectrophotometer, PG Instruments Ltd. UK)^66^.

To determine H_2_O_2_ concentration, leaf samples (1 g) were ground in 9 mL of normal saline solution (4.5 g NaCl added in 500 mL ddH_2_O) followed by centrifugation 10,000×*g* for 10 min. Three tube types were prepared, namely empty, standard and sample tubes. Briefly, reagent 1 and 2 (1.0 mL) in all tubes, H_2_O (0.1 mL) in empty tube, standard solution (0.1 mL) in standard tube, sample (0.1 mL) in sample tube was added. The absorbance was taken at 405 nm with spectrophotometer according to H_2_O_2_ determination kit (Nanjing Jiancheng Biology Co., Ltd.).

To determine electrolyte leakage (EL), fully expanded leaves from top of the plant canopy were taken followed by cutting into minor slices (5–6 mm length), placed in sterilized test tubes having 8 mL distilled water, incubated and transferred to water bath for 12 h prior to measuring the initial electrical conductivity (EC_1_). After measuring the initial EC_1_, samples were autoclaved at 121 °C for 20 min followed by cooling down to 25 °C to measure the final electrical conductivity (EC_2_)^67^. To measure the electrolyte leakage, a pH/conductivity meter (INCO-LAB Company, Kuwait) was used, then the following equation for EL calculation was applied:

2 $$\mathrm{EL }= (\mathrm{EC}1/\mathrm{EC}2) \times 100$$

To determine antioxidant activities, 0.5 g leaves were ground using a tissue grinder in 8 mL of cooled phosphate buffer (pH 7.0, containing 1% (w/v) polyvinylpyrrolidone) in test tubes. The homogenate was centrifuged at 15,000 rpm for 20 min at 4 °C. The supernatant was used for assays of enzymes activity. The activity of catalase (CAT) and peroxidase (POD) was measured by using the method of Maehly^68^. The reaction solution (3 mL) contained 0.1 mL standard enzyme extract, 15 mM H_2_O_2_ and 50 mM phosphate buffer (pH 7.0). The absorbance was taken at 240 nm with the spectrophotometer. The POD reaction solution (3 mL) contained 0.1 mL enzyme extract, 50 mM sodium acetate buffer (pH 5.0), 40 mM H_2_O_2_ and 20 mM guaiacol. The absorbance was taken at 470 nm. The superoxide dismutase (SOD) reaction solution (3 mL) contained 1.3 µM riboflavin, 50 µL enzyme extract, 50 µM nitro blue tetrazolium (NBT dissolved in ethanol), 13 mM methionine, 50 mM phosphate buffer (pH 7.8) and 75 nM EDTA^69^. The absorbance was taken at 240 nm.

### Statistical analysis

A three-way analysis of variance (ANOVA) was carried out, which compared the effects of two temperatures, two genotypes, and five GA_3_ levels. For the purpose of comparing the means of the different treatments (where *p* ≤ 0.05), a statistical programme Statistix 8.1 was used to run a test called Tukey's honest significant difference (HSD). Principal component analysis was then performed on the variables using XLSTAT version 2018. The Pearson (*n*) technique was used to arrive at the values of the correlation coefficient.

### Ethical declarations

This study was complied with the relevant institutional, national, and international guidelines and legislations. The permission was obtained for collection of tomato seeds from Vegetable Research Institute, Ayyub Agriculture Research Institute, Faisalabad, Pakistan.

## Conclusions

According to the findings of this research, applying GA_3_ to tomato plants by foliar spray might reduce the negative effects of heat stress on the plant and boost its physiological response as well as its growth. Due to the fact that foliar treatments of 25, 50, 75, and 100 mg L^−1^ GA_3_ differently affect separate components of plant growth and development, a certain concentration of GA_3_ may assist accomplish a specific target of thermotolerance. In general, an exogenous application approach of 75 mg L^−1^ GA_3_ has the potential to be an effective method for improving the overall plant health of tomato plants when heat stress is present. It is necessary to understand the molecular mechanism that are triggered by GA_3_ and that regulate stress-related features.

## Data Availability

All data generated or analysed during this study are included in this published article.
